# Ovarian Cancer in Child of Eight

**Published:** 1913-11

**Authors:** 


Ovarian Cancer in Child of Eight. Etienne and Aimes, *La Presse Med.*, May 14, 1913. Uterine hæmorrhage and a large, painless, mobile tumor were the principal symptoms. Mistologic examination showed epithelioma. The immediate progress was favorable but fears are entertained of recedive.

